# Cytoprotective Role of Omentin Against Oxidative Stress-Induced Vascular Endothelial Cells Injury

**DOI:** 10.3390/molecules25112534

**Published:** 2020-05-29

**Authors:** Nur Aqilah Binti Kamaruddin, Lai Yen Fong, Jun Jie Tan, Muhammad Nazrul Hakim Abdullah, Manraj Singh Cheema, Fahmi Bin Yakop, Yoke Keong Yong

**Affiliations:** 1 Department of Human Anatomy, Faculty of Medicine and Health Sciences, Universiti Putra Malaysia, UPM Serdang 43400, Selangor, Malaysia; aqikama05@gmail.com; 2 Department of Anatomy, Faculty of Medicine, Universiti Kebangsaan Malaysia, Jalan Yaacob Latif, Bandar Tun Razak, Cheras 56000, Kuala Lumpur, Malaysia; 3 Department of Pre-clinical Sciences, Faculty of Medicine and Health Sciences, Universiti Tunku Abdul Rahman, Kajang 43000, Selangor, Malaysia; fongly@utar.edu.my; 4 Advanced Medical and Dental Institute, Universiti Sains Malaysia, Bertam, Kepala Batas 13200, Penang, Malaysia; jjtan@usm.my; 5 Department of Biomedical Science, Faculty of Medicine and Health Sciences, Universiti Putra Malaysia, UPM Serdang 43400, Selangor, Malaysia; nazrulh@upm.edu.my (M.N.H.A.); manraj@upm.edu.my (M.S.C.); 6 Department of Basic Sciences and Oral Biology, Faculty of Dentistry, Universiti Sains Islam Malaysia, Kuala Lumpur 55100, Malaysia; fmie91@gmail.com

**Keywords:** omentin, oxidative stress, hydrogen peroxide, human umbilical veins endothelial cell, endothelial injury

## Abstract

Endothelial cell injury caused by reactive oxygen species (ROS) plays a critical role in the pathogenesis of cardiovascular diseases. Omentin, an adipocytokine that is abundantly expressed in visceral fat tissue, has been reported to possess anti-inflammatory and antidiabetic properties. However, endothelial protective effects of omentin against oxidative stress remain unclear. This study aimed to evaluate the protective effect of omentin against hydrogen peroxide (H2O2)-induced cell injury in human umbilical vein endothelial cells (HUVECs). Cytotoxicity and cytoprotective effects of omentin were evaluated using 3-(4,5-Dimethylthiazol-2-yl)-2,5-diphenyltetrazolium bromide (MTT) assay. The apoptotic activity of HUVECs was detected using Annexin-V/PI and Hoechst 33258 staining methods. Antioxidant activity of omentin was evaluated by measuring both reactive oxygen species (ROS) levels and glutathione peroxidase (GPx) activity. No cytotoxicity effect was observed in HUVECs treated with omentin alone at concentrations of 150 to 450 ng/ml. MTT assay showed that omentin significantly prevented the cell death induced by H_2_O_2_ (*p* < 0.001). Hoechst staining and flow cytometry also revealed that omentin markedly prevented H_2_O_2_-induced apoptosis. Moreover, omentin not only significantly inhibited ROS production (*p* < 0.01) but also significantly (*p* < 0.01) increased GPx activity in HUVECs. In conclusion, our data suggest that omentin may protect HUVECs from injury induced by H_2_O_2_.

## 1. Introduction

The blood vessel wall consists of a single layer of endothelial cells (EC), which plays important roles in hemostasis, vasodilatation, angiogenesis, vascular permeability, and the response of blood vessels to other physiological and pathological stimuli. EC possess both metabolic and synthetic functions and serve as the mechanical barrier between the circulating blood and vascular smooth muscle cells. By secreting a large variety of mediators such as growth factors, anti-thrombotic factors, inflammatory mediators, and others, EC are able to control a vast array of cellular functions throughout the body [1]. Alterations of endothelial cell structure and functions, hence contributing to the pathophysiology of vascular diseases. Endothelial cell injury is one of the initial events in the development of atherosclerosis [2] and oxidative stress is regarded as a hallmark of endothelial cell injury [3]. 

Oxidative stress is a result of an imbalance between the pro-oxidant and the antioxidant defense mechanisms in the body. An excessive amount of reactive oxygen species (ROS) causes severe damage to cellular structures and components including nucleic acids, proteins, and lipids, thereby leading to cell death [4]. It has been reported that exposure to excessive ROS is one of the main causes of endothelial cell injury and apoptosis [5,6]. Among ROS, hydrogen peroxide (H_2_O_2_) penetrates freely through the plasma membrane and damages neighboring cells as well as H_2_O_2_-producing cells. In view of this feature, H_2_O_2_ is extensively used to induce oxidative stress in many in vitro models [7,8]. Therefore, inhibition of H_2_O_2_-induced oxidative stress and injury in endothelial cells has been considered as a potential therapeutic strategy for cardiovascular diseases [9,10]. 

In physiological conditions, the level of intracellular ROS is tightly controlled by the antioxidant defense system in cells. ROS is directly detoxified or reduced into nontoxic substances through the action of nonenzymatic radical scavengers (e.g.; ascorbic acid and glutathione) and antioxidant enzymes (e.g., catalase, glutathione peroxidase (GPx), and superoxide dismutase (SOD)). High concentration of H_2_O_2_ is eliminated by glutathione peroxidase biochemically where glutathione peroxidase neutralizes H_2_O_2_ to water using two hydrogen atoms from two molecules of reduced glutathione (GSH) to form oxidized glutathione (GSSG) [11]. Thus, the glutathione system serves as an effective redox buffer to maintain low ROS levels in the cytosol and the balance between GSH and GSSG may reflect the redox status of the cell [12].

Omentin is an adipokine produced by visceral adipose tissue with insulin-sensitizing effects. Omentin is detectable in human plasma from 100 to 800 ng/ml [13]. The plasma level of omentin was found to be lowered in patients with obesity, impaired glucose tolerance, and type 2 diabetes mellitus, of which the diabetic patients had the lowest level of omentin, compared to control [13,14,15]. Omentin has also been reported to enhance vasodilation in isolated blood vessels and to reduce inflammatory responses in cultured endothelial cells [16,17]. Using a hind limb ischemia mouse model, omentin was demonstrated to enhance blood flow recovery and capillary density in ischemia limbs of mice. At the cellular levels, omentin increased differentiation of EC into vascular-like structures and lowered the apoptotic activity of EC via activation of the PI3K/Akt signaling cascade [18]. Omentin also ameliorates acute ischemic injury in the heart by suppressing cardiomyocyte apoptosis through the AMP-activated protein kinase (AMPK) and the Akt signaling pathways [19]. Although the roles of omentin in obesity-related metabolic and cardiovascular complications have been well studied, it remains unclear whether omentin can effectively protect the endothelium against injury evoked by oxidative stress. Therefore, the current study aimed to evaluate the protective effect of omentin in oxidative stress-induced endothelial cell injury. 

## 2. Results

### 2.1. Effect of Omentin and H_2_O_2_ in the Viability of Human Umbilical Vein Endothelial Cells (HUVECs) 

**Figure 1:**
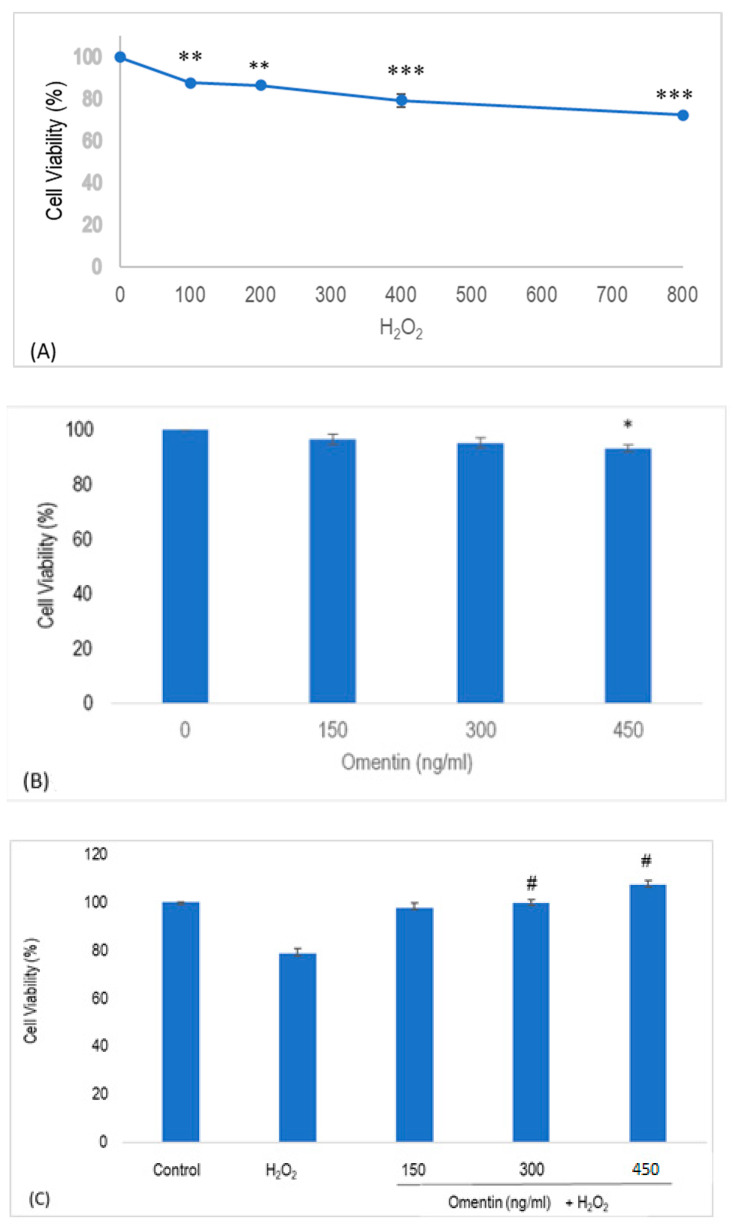
Cell viability as determined with 3-(4,5-Dimethylthiazol-2-yl)-2,5-diphenyltetrazolium bromide (MTT) assay. (**A**) Human umbilical vein endothelial cells (HUVECs) were incubated with of 0–800 µM H_2_O_2_ for 24 h. (**B**) The effect of omentin on the viability of HUVECs. (**C**) The protective effect of omentin in H_2_O_2_-induced injury. HUVECs were pretreated with media or 150–450 ng/ml omentin for 1h before exposure to 400 μΜ H_2_O_2_ for 4 h. Cell viability was determined using MTT assay. Values are mean ± SEM (*n* = 3). Data are expressed as means ± SEM (*n* = 3). * *p* < 0.05, ** *p* < 0.01, *** *p* < 0.001 compared to the control group, # *p* < 0.001 compared to H_2_O_2_ group.

As presented in Figure 1A, HUVECs proliferation was significantly inhibited in a concentration-dependent manner following the exposure of H_2_O_2_ from 100–800 µM for 4 h, compared to the control group (*p* < 0.01; Figure 1A). To ensure that the doses of omentin used do not cause cytotoxicity, its effects on the viability of HUVECs were examined in the present study. As presented in Figure 1B, 450 ng/ml of omentin significantly decreased the viability of HUVECs (*p* < 0.05) to 90% after 1 h. On the other hand, no cytotoxicity was observed in HUVECs treated with 150 and 300 ng/ml of omentin. In another set of experiments, 400 μM of H_2_O_2_ significantly lowered HUVEC viability (*p* < 0.001) to 78.6% (Figure 1C). As no significant difference was observed between the effects of 400 µM and 800 µM of H_2_O_2_ in HUVEC viability, 400 µM of H_2_O_2_ was chosen to be used in subsequent experiments. Further, pre-treatment with omentin (150, 300, and 450 ng/ml) significantly increased the viability of the H_2_O_2_-treated HUVECs where the survival rates were 97.9%, 99.7%, and 107.3%, respectively. These results suggest that omentin protects HUVECs from oxidative stress-related cellular injury.

### 2.2. Effect of Omentin on H_2_O_2_-Induced Apoptosis in HUVECs

**Figure 2:**
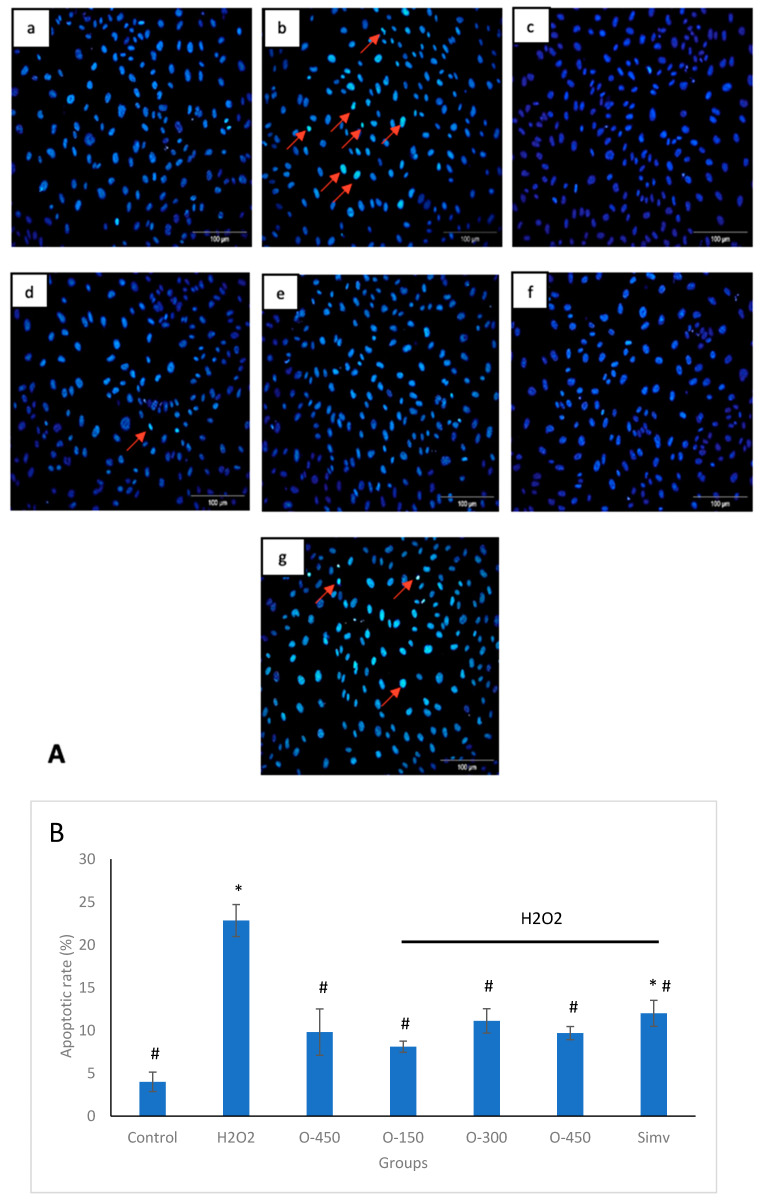
Effect of omentin on H_2_O_2_-induced HUVEC apoptosis. (**A**) HUVECs were treated with H_2_O_2_ in the presence or absence of omentin, and the cells were stained with Hoechst 33358. (**a**) Control, (**b**) 400 μM H_2_O_2,_ (**c**) 450 ng/ml omentin (**d**) 150 ng/ml omentin + 400 μM H_2_O_2_ (**e**) 300 ng/ml omentin + 400 μM H_2_O_2_ (**f**) 450 ng/ml omentin + 400 μM H_2_O_2_ and (**g**) Simvastatin (5 μM) + 400 μM H_2_O_2_. Red arrows indicate apoptotic cells; magnification, 200×. (**B**) Apoptotic rate was analyzed using Image J software. Data are expressed as mean ± SEM (*n* = 3). * *p* < 0.05 compared to control group, # *p* < 0.001 compared to H_2_O_2_ group. O-150—Omentin 150 ng/ml; O-300—Omentin 300 ng/ml; O-450—Omentin 450 ng/ml; Simv—Simvastatin 5 μM.

Nuclear morphological changes were evaluated using a fluorescence microscope and apoptotic cells are indicated with arrows in Figure 2B. Apoptotic cells are characterized by highly fluorescent condensed chromatin. After treating with 400 μM H_2_O_2_ for 4 h, HUVECs showed condensed, fragmented, and small nuclei, which is a typical apoptotic morphology, in comparison to the nuclear size of the control group (Figure 2a). This was attenuated by pre-treatment with omentin (Figure 2d–f). The images were further evaluated using Image J software. Based on the data obtained, H_2_O_2_ significantly increased the apoptotic rate of HUVECs to 22.85%, compared to 4.01% seen in the control group. Interestingly, all concentrations of omentin significantly reduced the apoptotic rate where a highest reduction rate was caused by 150 ng/ml of omentin (78.25% reduction). There was no significant difference between the effects of all the concentrations of omentin.

**Figure 3:**
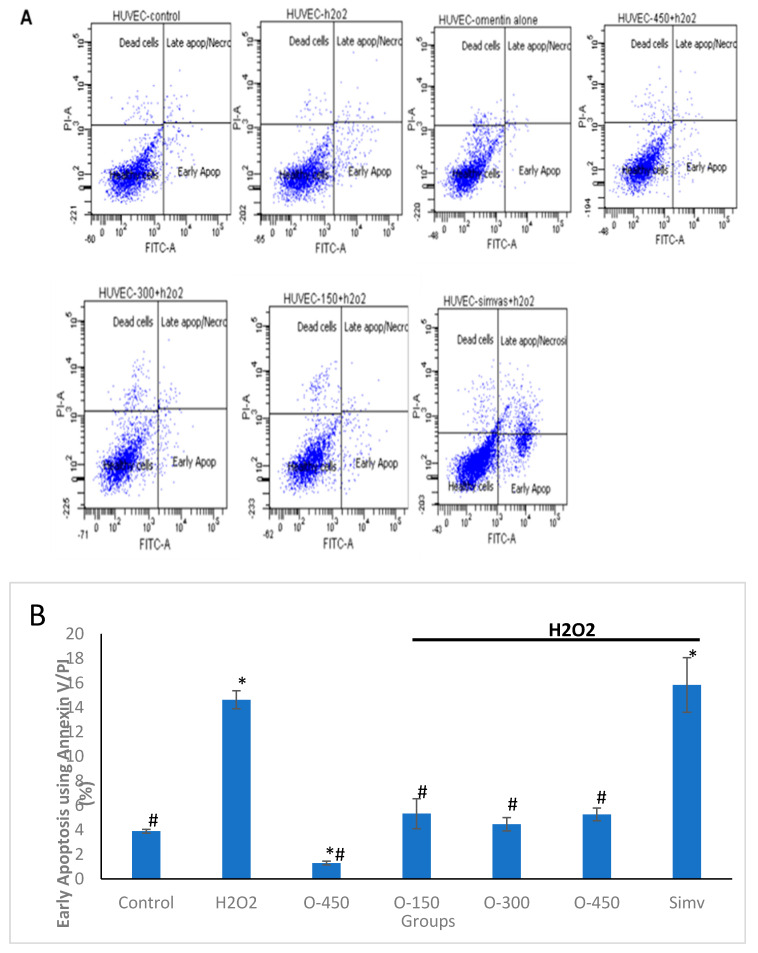
Omentin suppresses the apoptosis of HUVECs induced by H_2_O_2_. HUVECs pre-treated with omentin for 1 h were exposed to 400 μM H_2_O_2_ for 4 h, and then the cells were labeled with Annexin-V/PI staining and analyzed with flow cytometry. (**A**) Representative flow cytometry density plots of the control group, the H_2_O_2_ group and cells pre-treated with various concentrations of omentin or simvastatin. (**B**) Fluorescence intensities of HUVECs measured in the density plots. The data shown are mean ± SEM (*n* = 3). * *p* < 0.001 vs control. # *p* < 0.001 vs H_2_O_2_. Simv—simvastatin.

To further confirm this observation, flow cytometry with Annexin V-FITC/PI double staining was performed (Figure 3A,B). In line with the results of Hoechst staining assay, Annexin V-FITC/PI double staining showed a significant increase in cell apoptosis in the H_2_O_2_ group (14 ± 0.7%), compared to the control group (3.9 ± 0.15%). However, pre-treatment with different concentrations of omentin (150 ng/ml, 300 ng/ml and 450 ng/ml) for 1 h significantly decreased the percentage of early apoptosis to 5.3 ± 1.23%, 4.5 ± 0.55% and 5.3 ± 0.52%, respectively. Meanwhile, treatment with omentin (450 ng/ml) alone had no significant effect on cell apoptosis. These results demonstrate that omentin protects HUVECs from oxidative stress-induced apoptosis in vitro.

### 2.3. Antioxidant Effect of Omentin in H_2_O_2_-Induced ROS Production in HUVECs

**Figure 4:**
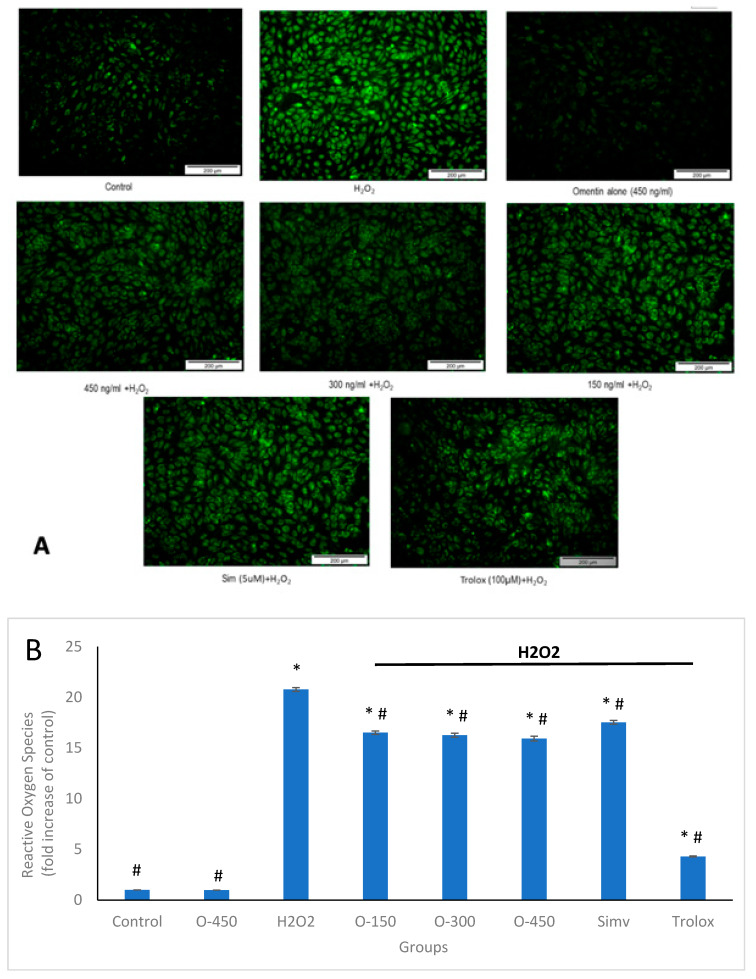
Effects of omentin on H_2_O_2_-induced reactive oxygen species (ROS) production in HUVECs. HUVECs were pre-treated with or without omentin for 1 h before stimulated with 400 µM H_2_O_2_ for another 1 h. The cells were then stained with 10 µM of DCFH-DA at 37 °C for 20 min. (**A**) Representative fluorescent images indicating ROS production in HUVECs. (**B**) Intracellular ROS levels were measured using a spectrofluorometer. Data are expressed as mean ± SEM (*n* = 3). * *p* < 0.001 compared to control group, ^#^ *p* < 0.001 compared to H_2_O_2_ group. O-150—Omentin 150 ng/ml; O-300—Omentin 300 ng/ml; O-450—Omentin 450 ng/ml; Simv—Simvastatin 5 μM; Trolox—Trolox 100 μM.

Exogenous addition of H_2_O_2_ induces intracellular ROS production, which affects cell metabolism [20]. To investigate whether inhibition of apoptosis by omentin is related to intracellular production of ROS, we measured intracellular ROS levels with DCFH-DA fluorescent dye using fluorescence microscopy and spectrofluorometry. As presented in Figure 4A, DCF fluorescence in HUVECs exposed to H_2_O_2_ was increased, compared to the control group. Pre-treatment with omentin evidently attenuated the DCF fluorescence elicited by H_2_O_2_. Spectrofluorometry showed that the production of ROS in HUVECs exposed to H_2_O_2_ alone was increased to about 20-folds, compared to the control group (Figure 4B). Pre-treatment with omentin and simvastatin (positive control) at indicated concentrations significantly reduced the level of ROS production, in agreement with the data obtained with fluorescence microscopy. In addition, omentin alone caused no significant changes in the ROS levels of HUVECs.

### 2.4. Effect of Omentin in H_2_O_2_-Induced Decreased GPx Activity in HUVECs

**Figure 5:**
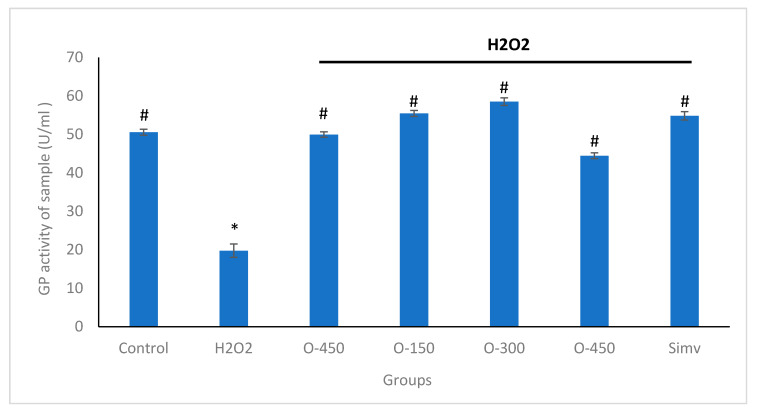
Omentin restores H_2_O_2_-induced glutathione peroxidase (GPx) depletion in HUVECs. HUVEC were treated with different concentrations of omentin (150, 300, and 450 ng/mL) for 1 h followed by 400 μM H_2_O_2_ for 4 h. GPx activity in the cells was measured using a spectrophotometer. Data are mean ± SD. from three independent experiments. * *p* < 0.01 compared control; # *p* < 0.01 compared H_2_O_2_ alone. O-150—Omentin 150 ng/ml; O-300—Omentin 300 ng/ml; O-450—Omentin 450 ng/ml; Simv: Simvastatin 5 μM.

To protect cells from the damage caused by ROS, excessive ROS is removed by several intracellular defense mechanisms that involve antioxidant enzymes such as GPx. The activity of GPx is shown in Figure 5. The activity of GPx was significantly decreased in H_2_O_2_ group (19.74 U/ml ± 1.76), compared to the control group (50.57 U/ml ± 0.773). Pre-treatment with omentin at concentrations of 150, 300, 450 ng/mL significantly elevated the activity of GPx to 55.44 U/ml ± 0.76, 58.5 U/ml ± 0.99, 44.40 U/ml ± 0.76, respectively. Simvastatin, at 5 µM, also augmented GPx activity to 54.71 U/ml ± 1.1. In addition, omentin alone was found to increase the activities of the antioxidant enzyme, compared to the H_2_O_2_ group.

## 3. Discussion

Oxidative stress is mainly caused by excessive accumulation of ROS that include H_2_O_2_, superoxide anions (O_2_^−^), and hydroxyl radicals (·OH). In physiological conditions, ROS are continuously produced in cells as products of cellular oxidation-reduction processes and they are crucial for biophylaxis. At high concentrations, ROS cause severe damage to cellular structures and components including nucleic acids, proteins, and lipids, thereby leading to cell death [21]. 

Omentin is a novel adipose tissue-derived adipokine and is highly expressed in visceral adipose tissue. Previous studies showed that omentin stimulates vasodilation in isolated blood vessels and suppresses cytokine-stimulated inflammatory responses in cultured endothelial cells [16,17]. Moreover, omentin promotes endothelial cell function and revascularization in an ischemic mice model through the Akt-eNOS signaling pathway [18]. Omentin has also been demonstrated to inhibit TNF-α-induced VCAM-1 expression via inactivation of p38 and JNK, with a concomitant inhibition of superoxide production [22]. Therefore, in the present study, we examined the effects of omentin against oxidative stress in HUVECs. 

In this study, we used H_2_O_2_ to induce a stable response of oxidative stress in HUVECs and this enables us to reproduce similar responses in all the experiments. H_2_O_2_ diffuses freely into and out of the cells and modulates multiple cellular processes such as cell proliferation, signal transduction, gene expression, DNA damage, apoptosis, and necrosis [23]. Firstly, we determined the toxicity of omentin in HUVECs using MTT assay. We showed that omentin alone did not affect the viability of HUVECs, whereas pretreatment of omentin significantly augmented H_2_O_2_-induced decreased cell viability (Figure 1A). In addition, flow cytometry showed that the percentage of apoptosis was increased in HUVECs after exposure to 400 μM H_2_O_2_ for 4 h. This was attenuated by omentin (Figure 2). To confirm the effect of omentin in H_2_O_2_-induced apoptosis in HUVECs, Hoechst 33358 was used to stain the DNA of the cells. Apoptotic cells were easily identified through their bright, condensed and fragmented DNA, while non-apoptotic cells showed chromatin of an even, flat disc-like morphology. These results suggest that omentin inhibits the apoptosis of endothelial cells exposed to oxidative stress. 

ROS are free radicals present in all types of vascular cells and hence, play an important role in endothelial injury [24]. ROS are produced by endothelial cells, smooth muscle cells, adventitial fibroblasts, and inflammatory cells. Basal levels of ROS contribute to normal vascular function whereas high concentrations of these chemical species provoke cell injury. Numerous studies suggested that ROS are involved in vascular remodeling and endothelial dysfunction [4,25]. Overproduction of ROS stimulates formation of lipid hydroperoxides and reactive lipid electrophiles such as 4-hydroxy-2-nonenals (4-HNE). 4-HNE is a major bioactive marker of lipid peroxidation also a growth-regulating factor and signaling molecule involved in cell proliferation, differentiation, and apoptosis interacting with cytokines in a concentration-dependent manner [26]. Accumulation of 4-HNE results in cellular dysfunction and oxidative damage in organelles such as mitochondria and the endoplasmic reticulum, leading to apoptosis [27]. Previous study showed that upon 4-HNE treatment in human microvascular endothelial cells enhanced the production of ROS [28]. Thus, it is suggested that 4-HNE plays a significant role in oxidative stress-induced cell injury. In our study, the production of ROS in HUVECs was examined using DCFDA and fluorescence microscopy. The result demonstrated that incubation of HUVECs with H_2_O_2_ increased ROS production, and this was significantly suppressed by omentin. The fluorescent staining of DCF also showed a consistent result (Figure 4A). An increase in ROS production could be due to the reaction between H_2_O_2_ and peroxidase, such as myeloperoxidase, to form highly reactive molecules like HOCl [29]. H_2_O_2_ activates a number of oxidant-producing enzymes that result in ROS production in vascular cells, for instance, activation of NADPH oxidase leads to generation of O_2_^−^ [29]. Our results showed that omentin suppressed H_2_O_2_-induced ROS production in HUVECs, and this could be partly attributed to the capability of omentin to scavenge free radicals or interfere with the activity of oxidant-producing enzymes stimulated by H_2_O_2_. These findings also suggest that a significant inhibition of H_2_O_2_-induced ROS production may contribute to restoring the viability of HUVECs. Moreover, this effect might be, at least in a part, due to the lipid peroxidation chain reactions that might generate HNE acting as a second messenger of free radicals. However, it is strongly encouraging to further research the involvement of 4-HNE in the current experimental setting. 

GPx is a crucial enzyme in cellular antioxidant systems and plays critical roles in the elimination of excessive ROS in living organism. GPx catalyzes the reduction of hydrogen peroxide to water and oxygen using two hydrogen atoms from two molecules of GSH to form GSSG. Inadequate elimination of ROS results in oxidative stress that may cause severe metabolic malfunctions and damage to biological macromolecules. In the present study, H_2_O_2_ significantly decreased the antioxidant enzyme levels in HUVECs. The results indicate that the production of high levels of ROS triggered by H_2_O_2_ may decrease antioxidant enzymatic activities in the cells. Pre-treatment with omentin significantly increased intracellular GPx activity. We suggest that omentin may eliminate intracellular ROS by activating the intracellular antioxidant system. In short, omentin suppresses oxidative stress partially by decreasing the generation of ROS and this could be due to the ability of omentin to enhance the intracellular antioxidant system, for example, the activity of GPx. The protective effect of omentin against oxidative stress could underlie its inhibitory effect in H_2_O_2_-induced HUVEC injury, which in turn increases the viability and functionality of HUVECs.

## 4. Materials and Methods 

### 4.1. Chemicals and Reagents

Recombinant human omentin was purchased from Biovendor (Candler, NC, USA). EndoGRO Basal Medium supplemented with kit, H_2_O_2_ and 2’,7’-dichlorodihydrofluorescein diacetate (H2DCFDA) were purchased from Merck Millipore (Darmstadt, Germany). 3-(4,5-dimethylthiazol-2-yl) -2,5-dephenyltetrazolium bromide (MTT) and Hoechst 33358 were purchased from Sigma Co. (St. Louis, MO, USA). 2.5% Trypsin-EDTA solution 10× was purchased from Santa Cruz Biotechnology (Santa Cruz, CA, USA). Annexin V/PI assay kit and GPx assay kits were purchased from Trevigen (Gaithersburg, MD, USA). All the other chemicals used were of analytical grade.

### 4.2. HUVECs Culture

Human umbilical vein endothelial cells (HUVECs) were purchased from Merck Millipore (Darmstadt, Germany) and the cells were cultured with EndoGRO-LS Complete Culture Media Kit. The cell suspension from the newly purchased vial were grown into eight 25 cm^2^ flasks. Cells were maintained in a humidified incubator with 5% CO_2_ at 37 °C and the medium was changed every two days till the cultures reached 90% confluence. The cells were trypsinized using 2.5% Trypsin-EDTA solution 10x, centrifuged (1200 rpm for 10 min) and subcultured into five 25 cm^2^ flasks. Passages 3–4 were used in all the experiments to maintain cell morphology and biological properties.

### 4.3. Treatment Protocols

For all experiments, otherwise specified in the protocol, HUVECs were cultured to confluence in 96-well plates or 6-well plates. The cells were pre-treated with omentin (150, 300, and 450 ng/mL) for 1 h before exposure to H_2_O_2_ (400 μM) for 4 h. Following exposure to H_2_O_2_, the cells were harvested for further analysis.

### 4.4. Cell Viability Assay

Cell viability was assessed using MTT as previously described with some modifications [30]. Briefly, HUVECs were seeded at 2 × 10^4^ cells per well onto a 96-well plate. In order to determine the cytotoxic effects of omentin and H_2_O_2_, HUVECs were treated with 150–450 ng/ml of omentin for 1 h or 0–800 µM H_2_O_2_ for 4 h. All treatments were prepared in 100 µL of media. Following treatments, 50 µL of MTT solution (2 mg/mL in PBS) was added into each well which contained 100 µL of treatment and incubated at 37 °C for another 4 h. The MTT solution was removed and 100 µL of Dimethyl Sulfoxide (DMSO) was added to dissolve the purple formazan. The optical density (OD) was measured using a microplate reader at 570 nm (Infinite M200, TECAN, Mannedorf, Switzerland). In another set of experiments that was designed to confirm the cytoprotective effect of omentin, HUVECs were treated with omentin (150–450 ng/mL) for 1 h before stimulated with H_2_O_2_ (400 µM) for 4 h. 100 µL of omentin was first added to the well and incubated for 1 h. Then, the media was removed and 100 µL of H_2_O_2_ was added into the well for 4 h in the cytoprotective assay. Each set of experiments was performed in triplicate. The cell viability was expressed in percentage.

### 4.5. Hoechst 33358 Fluorescence Staining

HUVEC were seeded at 2 × 10^4^ cells per well onto a 96-well plate and grown for overnight. After treatment, the cells were washed twice with 100 µL of cold PBS and fixed with 100 µL cold methanol and acetic acid at a ratio of 3:1 (*v/v*) for 30 mins in dark. The fixed cells were stained with 20 µL of Hoechst 33358 (1 mg/ml) for another 30 mins in dark [31]. The cells were then observed using a fluorescence microscope (Microscope, Olympus DX51, Tokyo, Japan; Camera, Olympus DP72). Normal HUVECs exhibited a normal nuclear size and had a uniform fluorescence intensity, whereas apoptotic cells exhibited increased membrane permeability, shrinkage, and nuclei that are condensed. The apoptotic cells were counted in 5 different areas of 3 visual fields for each group using Image J software and expressed as apoptotic rate. The apoptotic rate (percentage of apoptotic cells) was calculated with a formula: the number of apoptotic cells / total cells counted × 100.

### 4.6. Flow Cytometric Analysis of Apoptosis

Annexin-V/PI assay was performed using BD Annexin V-FITC Assay kit according to the manufacturer’s instructions. Briefly, HUVECs cells were seeded at 1  ×  10^6^ per well in a 6-well plate and incubated for 24  h. The cells were treated with omentin (150, 300, and 450 ng/mL) for 1 h and induced with H_2_O_2_ (400 µM) for another 4 h. After treatments, cells were washed once with 1X PBS, trypsinized and centrifuged at 1000 rpm for 5 minutes. The cell pellets were washed twice with cold PBS and resuspended in 100 µL of 1× Annexin-binding buffer. The suspension was transferred to a 1.5-mL tube, and 5 µL of Annexin V and 5 µL of PI were added to 100 µL of cell suspension. The cells were incubated in dark at room temperature for 15 minutes and 400 µL of 1× Annexin-binding buffer was added. After mixing gently, the fluorescence intensity was detected with a flow cytometer (BD Biosciences, San Diego, CA, USA). Data were analyzed using BD FACSDiva Software version 6.1.3. Approximately 1–5 × 10^5^ cells were analyzed in each sample.

### 4.7. Measurement of Intracellular Reactive Oxygen Species (ROS)

The formation of ROS was measured using a ROS-sensitive dye, 2,7-dichlorodihydro-fluorescein diacetate (DCF-DA, Invitrogen, Carlsbad, CA, USA), as an indicator. Cells were seeded at 2 × 10^4^ cells/well in a black and clear bottom 96-well plate containing 100 µL of culture medium and incubated at 5% CO_2_ and 37 °C overnight. The cells were pretreated with omentin (150–450 ng/ml) or 2 positive controls ((simvastatin (5µM) and Trolox (100µM)) for 1 h. Next, the media were removed, and the cells were washed twice with PBS, and this was followed by stimulation with 400 uM H_2_O_2_ for 1 h. The cells were then incubated with 5 μM 2′,7′-dichlorofluorescin diacetate (DCFDA) for 30 min at 37 °C [32]. The fluorescent product formed was quantified with a spectrofluorometer at the 480/520 nm. Then, the fluorescent cells were washed twice with PBS and observed using a fluorescence microscope (Microscope, Olympus DX51; Camera, Olympus DP72).

### 4.8. Measurement of the Intracellular GPx

A cellular GPx assay kit (Trivigen, Gaithersburg, MD, USA) was used to measure GPx activity. Briefly, media in a flask were discarded after treatments and HUVECs were washed with 1X PBS. The cells were harvested by scrapping the cell monolayer in PBS. The cells were then centrifuged at 400× *g* for 10 min. The cell pellets were washed with 1X cold PBS and resuspended in an ice-cold 1X assay buffer (HT Glutathione Peroxidase Assay Kit, Trivigen, Gaithersburg, MD, USA) containing 0.4 mM Phenylmethylsulfonyl fluoride (PMSF) and 1% Triton X-100. The cell suspension was incubated on ice and vortexed periodically for 30 min. The cell lysate was centrifuged at 10,000× *g* for 15 min at 4 °C, and the supernatant was collected and stored at −80 °C until the assay was performed. The protein concentration of the cleared cell lysate was determined with a nanodrop spectrophotometer. GPx activity in the supernatant was determined according to the protocol provided by the manufacturer. 

### 4.9. Statistical Analysis

All data were presented as means ± SEM of three independent experiments carried out in triplicates. Statistical analysis was performed using one-way analysis of variance (ANOVA) Tukey’s post hoc test in IBM SPSS statistics version 22.0. A *p*-value of less than 0.05 (*p* < 0.05) was regarded as statistically significant.

## 5. Conclusions

Taken together, our present work shows that omentin possesses a protective effect against H_2_O_2_-induced cytotoxicity and apoptosis in HUVECs. In addition, omentin inhibits intracellular ROS formation and enhances the antioxidant defense system in HUVECs exposed to oxidative stress-induced injury. As oxidative stress-stimulated endothelial cell injury plays an important role in the development of atherosclerosis, the findings of the present study may shed light on the pharmacological basis and the clinical application of omentin in treatment of atherosclerosis.
